# The role of PKC/PKR in aging, Alzheimer's disease, and perioperative neurocognitive disorders

**DOI:** 10.3389/fnagi.2022.973068

**Published:** 2022-09-12

**Authors:** Wenping Lu, Sailan Tang, Ao Li, Qiuyue Huang, Mengyun Dou, Ye Zhang, Xianwen Hu, Raymond Chuen Chung Chang, Gordon Tin Chun Wong, Chunxia Huang

**Affiliations:** ^1^Department of Anesthesiology, The Second Affiliated Hospital of Anhui Medical University, Hefei, China; ^2^Key Laboratory of Anesthesiology and Perioperative Medicine of Anhui Higher Education Institutes, Anhui Medical University, Hefei, China; ^3^Scientific Research and Experiment Center of the Second Affilliated Hospital of Anhui Medical University, Hefei, China; ^4^The Second Clinical Medical College of Anhui Medical University, Hefei, China; ^5^Laboratory of Neurodegenerative Diseases, School of Biomedical Sciences, LKS Faculty of Medicine, The University of Hong Kong, Pokfulam, Hong Kong SAR, China; ^6^State Key Laboratory of Brain and Cognitive Sciences, The University of Hong Kong, Pokfulam, Hong Kong SAR, China; ^7^Department of Anaesthesiology, LKS Faculty of Medicine, The University of Hong Kong, Pokfulam, Hong Kong SAR, China

**Keywords:** perioperative neurocognitive disorders, autophagy, protein kinase C, double-stranded RNA (dsRNA)-dependent protein kinase, laparotomy

## Abstract

**Background:**

The incidence of perioperative neurocognitive disorders (PNDs) is reportedly higher in older patients. Mitochondrial and synaptic dysfunctions have consistently been demonstrated in models of aging and neurodegenerative diseases; nonetheless, their role in PND is not well understood.

**Methods:**

The Morris water maze and elevated plus maze tests were used to assess the learning and memory abilities of both C57BL/6 and 3×Tg-AD mice of different ages (8 and 18 months). PND was induced by laparotomy in C57BL/6 mice and 3×Tg-AD mice (8 months old). Markers associated with neuroinflammation, mitochondrial function, synaptic function, and autophagy were assessed postoperatively. The roles of protein kinase C (PKC) and double-stranded RNA-dependent protein kinase (PKR) were further demonstrated by using PKC-sensitive inhibitor bisindolylmaleimide X (BIMX) or PKR^−/−^ mice.

**Results:**

Significant cognitive impairment was accompanied by mitochondrial dysfunction and autophagy inactivation in both aged C57BL/6 and 3×Tg-AD mice. Laparotomy induced a significant neuroinflammatory response and synaptic protein loss in the hippocampus. Cognitive and neuropathological changes induced by aging or laparotomy were further exacerbated in 3×Tg-AD mice. Deficits in postoperative cognition, hippocampal mitochondria, autophagy, and synapse were significantly attenuated after pharmacological inhibition of PKC or genetic deletion of PKR.

**Conclusions:**

Our findings suggest similar pathogenic features in aging, Alzheimer's disease, and PND, including altered mitochondrial homeostasis and autophagy dysregulation. In addition, laparotomy may exacerbate cognitive deficits associated with distinct neuronal inflammation, mitochondrial dysfunction, and neuronal loss independent of genetic background. The dysregulation of PKC/PKR activity may participate in the pathogenesis of these neurodegenerative diseases.

## Introduction

For over half a century, it has been observed that some patients experience impairment in memory, attention, action, and perception after surgery, a condition previously known as postoperative cognitive dysfunction (POCD) but now termed perioperative neurocognitive disorder (PND). A systematic review of 274 studies has reported that the overall postoperative incidence at 1–3 months is around 29.0% (Borchers et al., 2021). It is well established that delayed neurocognitive recovery can have a long-term impact on daily living function and even mortality (Schwarz et al., 2013). A study reported that the incidence of dementia was 30.8%, and detectable cognitive impairment was 30.8% in elderly patients after coronary artery bypass graft at 7.5 years postoperatively. Patients with detectable impairment at 3 and 12 months postoperatively had increased mortality (Evered L.A. et al., 2016). Due to the pathological similarities between PND and Alzheimer's disease (AD), Aβ, tau, and neuroinflammation have been considered predictors of the development of PND (Evered L. et al., 2016). Interestingly, in the absence of clinically detectable AD symptoms, patients with AD neuropathology may be more susceptible to a postoperative cognitive decline (Evered L. et al., 2016).

The protein kinase C (PKC) family of serine/threonine kinases regulates diverse cellular functions for cell survival, proliferation, and death. Human PKC isoforms include PKCα, β, δ, ε, γ, and ζ, and imbalances in their expression and activities have been implicated in the pathophysiology of AD, diabetes, and cancers (Newton, 2018). In the central nervous system (CNS), these enzymes play critical roles in learning and memory, among other important functions (Sun and Alkon, 2014). Although the activation of some PKC isoforms, such as PKCα and ε, has been shown to regulate nonamyloidogenic pathways and Aβ degradation, it is unclear whether other PKC isoforms are involved in β-amyloid precursor protein (APP) processing and the pathogenesis of AD. In this regard, PKCδ reportedly governs cellular homeostatic responses, such as autophagy and apoptosis, against hypoxic stress through PKCδ/JNK1-mediated signaling pathways (Chen et al., 2009). PKCδ also plays an important role in exacerbating AD pathogenesis and may be a potential therapeutic target in AD. Importantly, in a transgenic AD mouse model, inhibition of PKCδ by rottlerin has been shown to markedly reduce the expression of β-site APP-cleaving enzyme I (BACE1, a novel aspartyl protease in Aβ genesis), Aβ levels, and plaque formation and improves cognitive deficits (Du et al., 2018).

Double-stranded RNA-dependent protein serine/threonine kinase (PKR) is a ubiquitous eukaryotic initiation factor 2α kinase that inhibits translation involving memory formation and consolidation. Current evidence suggests that PKR accumulates in the brain and cerebrospinal fluid in patients with AD and mild cognitive impairment, leading to TNFα and IL1-β production (Hugon et al., 2017). In addition, PKR contributes to inflammatory and immune dysfunction through mitogen-activated protein kinases, interferon regulatory factor 3, nuclear factor κB, apoptosis, and autophagy pathways (Kang and Tang, 2012). In a previous study, we demonstrated that PKR signaling participates in the development of PND with defective mitophagy in aged mice (Wang et al., 2022).

Given that PKC and PKR are implicated in the pathogenesis of Alzheimer's disease and advanced age is a risk factor for both AD and PND, we hypothesized that age-related changes in PKC and PKR activities are involved in the development of PND and may account for the increased susceptibility of the elderly. We used wild-type transgenic AD mice to compare age-related changes in PKC/PKR expression and mitochondrial function. Finally, we demonstrated the restorative effects of PKC inhibition PKR knockout on postoperative cognitive changes.

## Materials and methods

### Animals

Male C57BL/6 mice, 3×Tg-AD mice (triple transgenic B6; 129-*Psen*^1*tm*1*Mpm*^ Tg (APPSwe, tauP301L)1Lfa/J), and PKR^−/−^ mice were obtained from the Laboratory Animal Unit of Anhui Medical University and the University of Hong Kong. All experimental protocols and animal handling procedures were approved by the Faculty Committee on the Use of Live Animals in Teaching and Research at The University of Hong Kong (CULATR Ref. No. 3437-14) and the Ethics Committee for the use of experimental animals in Anhui Medical University (reference number LLSC20190765; date of approval 26/10/2019). The animals were randomly divided into specific groups depending on the protocols. The mice were bred and housed in a temperature- and humidity-controlled room. All animals had free access to food and water. Acclimatization for 1 week was completed before experiments. All behavioral tests were conducted from 09:00 to 12:00 P.M.

### Laparotomy

Bisindolylmaleimide X (hydrochloride) (BIMX) (Sigma, USA), a selective PKC inhibitor, was administered intraperitoneally at 30 min before laparotomy for the treatment groups. A laparotomy was performed under sevoflurane (Sevorane™, Abbott, Switzerland) anesthesia, induced *via* a rodent inhalation anesthesia apparatus (Harvard, USA), as previously described (Huang et al., 2018). The intestine was exposed through a longitudinal midline incision and rubbed vigorously for 30 s. After 1-min exposure, it was replaced in the abdominal cavity. Following sterile chromic gut sutures (4-0, PS-2; Ethicon, USA), the animals were returned to their cage for recovery. The entire procedure was completed within 15 min, and anesthesia was discontinued immediately after performing the last stitch. The control mice were exposed to sevoflurane only for 15 min.

### Morris water maze test

To assess allocentric navigational abilities, the Morris water maze (MWM) test was performed from postoperative days 6–12. This test required a circular tank (35 cm in depth with a diameter of 120 cm) containing a floating platform (7 cm high), conspicuous visual cues, and a video recording system (Debut Professional, NCH Software, Australia). The pool was divided into four quadrants, each with a specific visual cue. The platform was stabilized in the middle of one quadrant 0.7 cm below the water. The entire test consisted of a hidden platform training spanning over 6 days and the probe test without the platform. In each trial during training, the mice were randomly placed into each quadrant to search for the invisible platform. If the animal failed to reach the platform by itself within 60 s, it would be guided to the platform for a 20-s stay. The probe test was performed 24 h after the last training trial. The animals were placed in the opposite quadrant for 60 s. Finally, the learning curve was calculated by the accumulated escape latency in the training phase, and the number of platform crossings was used to assess spatial memory in the probe test.

### Elevated plus maze test

This test was used to evaluate the changes in anxiety-related behaviors in the mice. To assess the spontaneous activity and anxiety, an elevated plus maze test was performed on postoperative day 13. The apparatus consisted of two open and closed arms of the same size (35 cm length, 5 cm width, 15 cm height) and a central square connecting the arms. The apparatus was placed at a height of 40 cm from the ground (RWD Life Science, China). The animals were allowed to move freely in the elevated cross maze for 10 min, and their movements were recorded with a video camera. The number of crossings and time spent in the open arm were analyzed by PanLab Smart 3 software (Harvard, USA).

### Y-maze test

To assess the hippocampal-dependent spatial learning capacity, a modified Y-maze test was used (Huang et al., 2018). The device consisted of two black arms capable of delivering electric shocks at 2 Hz for 10s (40 ± 5 V) and one shock-free compartment. After habituation, each mouse was placed in one of the black compartments to assess spontaneous alternating behavior, and electric shocks were applied until it entered the shock-free compartment and stayed there for 30 s, which was then deemed a correct choice. Successful training was made with nine continuous correct choices. Each mouse was tested 10 times for the validation trial, following the same procedures as in the training trial. The number of incorrect choices and the time taken to enter the shock-free compartment (latency) were recorded.

### Transmission electron microscopy

The mice were killed 24 h after the elevated plus maze test. The hippocampi were sectioned (1 mm^3^) and fixed overnight in 2.5% glutaraldehyde at 4°C. After rinsing in PBS for 6 h at 4°C, the sections were post-fixed in 1% osmium tetroxide for 1 h at 4°C. Then, the sections were dehydrated with alcohol. After incubation in propylene oxide and epoxy resin for 2 h at room temperature, the sections were embedded in epoxy resin to obtain ultrathin sections (70 nm thick, Leica UC-7 microtome). The area and diameter of mitochondria (10,000×) were analyzed using ImageJ software (National Institutes of Health, USA). The number of mitochondria autophagy bodies (10,000×) was observed in at least 10 visual fields (including intact neuronal bodies) by using a JEM1400 electron microscope (JEOL, Japan).

### SDS-PAGE and western blot analysis

After transcardial perfusion, the left cerebral hemisphere was immediately frozen and embedded in an optimal cutting temperature (OCT) compound for further sectioning. The hippocampal tissues were dissected from the right hemisphere, and protein extracts were stored at −80°C. Whole-protein lysates were extracted using RIPA buffer containing protease and phosphatase inhibitors (Roche, Germany). The mitochondria and cytoplasmic fractions were collected using a mitochondria isolation kit (MITOISO1, Sigma, USA). The synaptosomal and cytosolic fractions were freshly prepared using Syn-PER^TM^ Synaptic Protein Extraction Reagent (Thermo Fisher Scientific, USA) plus protease and phosphatase inhibitors (Roche, USA), following the manufacturer's instructions.

Total lysates or cellular compartment fractions were subjected to 10 to 15% polyacrylamide gel electrophoresis and transferred onto PVDF membranes, as described previously (Huang et al., 2018). After blocking with 5% non-fat milk for 1 h at room temperature, the membranes were incubated overnight at 4°C with specific primary antibodies including Bax, Bcl-2, cleaved caspase 3, Pink1, Parkin, p62, LC3B, PKR, p-PKR, PKC, p-PKC, p-eif2α, eif2α (Cell Signaling Technology, USA), BDNF (Santa Cruz Biotechnology), and OXPHOS (Abcam, USA). After incubation with horseradish peroxidase-conjugated secondary antibodies (DAKO, Denmark) for 2 h at room temperature, the immunoreactive band signal intensity was subsequently visualized by chemiluminescence (SuperSignal^TM^ West Femto Maximum Sensitivity Substrate) (Thermo Fisher Scientific, USA). All immunoblots were normalized with β-actin or GAPDH (Sigma-Aldrich, USA) antibodies. The intensities of chemiluminescent bands were measured by ImageJ software (National Institutes of Health, USA).

### RNA isolation and real-time PCR

Total RNA from hippocampi and livers were extracted using Tri Reagent^®^ (MRC, USA) and purified using an Ambion^®^ DNA-free^TM^ DNA Removal Kit (Invitrogen, USA). After reverse transcription using a PrimeScript^TM^ Master Mix Kit (TAKARA, Japan), PCR was performed using an SYBR^®^ Premix Ex Taq^TM^ II Kit (TAKARA, Japan). The amplification conditions of IL-1β, IL-6, and IL-8 were previously described in the literature (Huang et al., 2018). In addition, monocyte chemoattractant protein-1 (MCP-1) was also detected by using the StepOnePlus^TM^ Real-Time PCR System (Applied Biosystems, USA) with the following sequence: F: 5′-TGCTGTCTCAGCCAGATGCAGTTA-3′, R: 5′-TACAGCTTCTTTGGGACACCTGCT-3′. The relative levels of cytokines were normalized to the endogenous reference glyceraldehyde-3-phosphate dehydrogenase (GAPDH) using the 2^−Δ*ΔCt*^ method.

### Dihydroethidium and JC-1 assay

To detect the level of reactive oxygen species (ROS), fresh frozen sections of the hippocampus were labeled with a dihydroethidium (DHE) fluorescence probe. Following activation in methanol for 20 min, 15-μm-thick sections were incubated with 10 μM DHE dye (Thermo Fisher Scientific, USA) for 20 min at 37°C. The images were then captured using a confocal microscope by excitation at 568 nm (Carl Zeiss LSM 900, Germany).

To assess for any changes in mitochondrial membrane potential, 5 μg of fresh mitochondrial proteins were incubated with a JC-1 dye (1 μg/ml, Thermo Fisher Scientific, USA) for 20 min at 37°C. The fluorescence intensity ratio of 525 nm/488 nm was measured by using a CLARIOstar instrument (BMG LabTech, Thermo Fisher Scientific, USA).

### Immunofluorescence staining and apoptosis assay

For immunofluorescence staining, 12-μm-thick coronal frozen sections were obtained from OCT embedded blocks (from−1.46 mm to−2.46 mm posterior to bregma). To block non-specific antibody binding, 10% normal goat serum was used after antigen retrieval with citrate buffer (0.01 M, pH 6.0) at 90°C for 15 min. Specific primary antibodies were applied at 4°C overnight as follows: IBA1 (Wako, Japan), GFAP (Sigma-Aldrich, USA), CD68 (Serotec, USA), cleaved caspase 3 (Cell Signaling Technology, USA), PSD95, and synaptophysin (Synaptic System, Germany). The sections were then incubated with specific Alexa Fluor 568 or 488 secondary antibodies (Invitrogen, USA) for 2 h at room temperature.

Apoptosis was detected using the TdT-mediated dUTP nick-end labeling (TUNEL) technique (*In Situ* Cell Death Detection Kit TMR red, Roche, USA). The nuclei were labeled with 4'-6-diamidino-2-phenylindole (DAPI) (3 μM, Sigma-Aldrich, USA). Photographs were observed under a laser scanning confocal fluorescent microscope (5×, 20×, and 40× oil immersion objectives) (Carl Zeiss LSM 900, Germany). *Z*-stack images were acquired at 1,024 × 1,024 resolution. All quantitative analyses were performed on at least three images acquired from three serial sections per animal.

### Statistical analyses

Using the statistic software GraphPad Prism 9.0 (Graph Pad Software Inc., USA), data from the Morris water maze were analyzed using a two-way analysis of variance (ANOVA), followed by the Tukey's *post hoc* test for repeated measures. Other data, such as elevated plus maze test, relative mRNA levels of cytokines, normalized band intensities in Western blot, and quantification of immunoreactivity, were analyzed by one-way ANOVA, followed by the Tukey's *post hoc* test or *t*-test. The normality of the data and homogeneity of group variances were assessed using the D'Agostino–Pearson omnibus normality test, Shapiro–Wilk normality test, and Kolmogorov–Smirnov test. A *P*-value < 0.05 was statistically significant.

## Results

### Mitochondrial characteristics, PKR and PKC activities, and cognitive performances during aging in both wild-type and 3×Tg-AD mice

**Figure 1:**
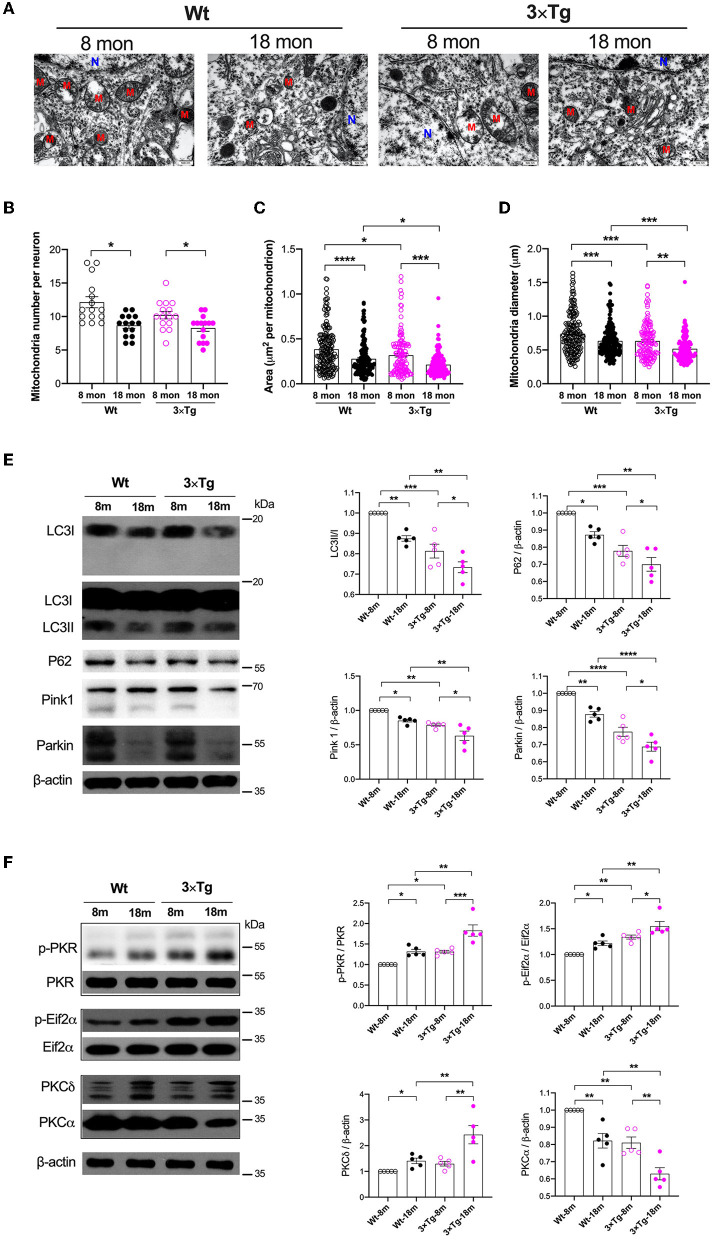
Variations of mitochondria and autophagy during aging in the wild-type and 3×Tg AD mice. **(A)** Representative electron microscopy photographs of mitochondria in the hippocampal neurons in different ages of wild-type and transgenic AD mice (N, nucleus; M, mitochondria; scale bar = 500 nm). **(B–D)** Quantification of mitochondrial number, area, and diameter in the hippocampal tissues from two strains of mice by electron microscopy, n = 182, 160, 138, 121 mitochondria from 15 neurons in five mice per group. **(B)**: F_(3, 56)_ = 8.787, *P* < 0.0001, *n* = 15; **(C)**: F_(3, 597)_ = 18.49, *P* < 0.0001, *n* = 121–182; **(D)**: F_(3, 597)_ = 20.05, *P* < 0.0001, *n* = 121–182. **(E)** In wild-type and 3×Tg AD mice, the relative expressions of autophagy-related proteins in the hippocampi were quantified by Western blotting analysis. LC3: F_(3, 16)_ = 24.74, *P* < 0.0001; P62: F_(3, 16)_ = 22.62, *P* < 0.0001; Pink1: F_(3, 16)_ = 17.26, *P* < 0.0001; Parkin: F_(3, 16)_ = 43.14, *P* < 0.0001. **(F)** Quantitative analysis of the relative expression of PKR, Eif2α, PKCδ, and PKCα in the hippocampal tissues in two strains of mice at different ages. p-PKR/PKR: F_(3, 16)_ = 20.58, *P* < 0.0001; p-Eif2α/Eif2α: F_(3, 16)_ = 18.22, *P* < 0.0001; PKCδ: F_(3, 16)_ = 10.51, *P* = 0.0005; PKCα: F_(3, 16)_ = 21.72, *P* < 0.0001. Wt, wild-type mice; 3×Tg, triple transgenic mice. Data are presented as mean ± SEM and were analyzed using one-way ANOVA using Tukey's *post hoc* test, *n* = 5, ^*^P < 0.05, ^**^P < 0.01, ^***^P < 0.001, ^****^P < 0.0001.

To assess age-related neuropathological changes, we first examined ultrastructural characteristics of neuronal mitochondria using a transmission electron microscope (Figure 1A). In both wild-type or 3×Tg-AD mice, the mitochondrial number, area, and diameter were significantly decreased in 18-month-old mice compared with their younger counterparts (mitochondrial number, 12.1 ± 3.2 vs. 8.7 ± 1.6 in wild-type mice, 10.2 ± 2.2 vs 8.3 ± 1.9 in 3×Tg-AD mice; mitochondrial area, 0.38 ± 0.24 μm^2^ vs. 0.28 ± 0.18 μm^2^ in wild-type mice, 0.32 ± 0.23 vs. 0.21 ± 0.13 μm^2^ in 3×Tg-AD mice; mitochondrial diameter, 0.74 ± 0.29 vs. 0.64 ± 0.23 μm in wild-type mice, 0.63 ± 0.25 vs. 0.52 ± 0.18 μm in 3×Tg-AD mice; Figures 1B–D). Levels of autophagy were reduced in the hippocampus during aging in both strains, as indicated by the significantly decreased expressions of autophagy-related proteins, including LC3, P62, Pink1, and Parkin. These protein levels were lower in 3×Tg-AD mice than in wild-type mice (Figure 1E). There were also changes in the activities of PKR and PKC during aging. Increased activity of PKR and Eif2α in terms of phosphorylation was observed in the hippocampus in both wild-type and 3×Tg-AD mice. With respect to the different isoforms, there was an enhancement in PKCδ and suppression in PKCα activity during aging in wild-type mice and 3×Tg-AD mice (Figure 1F).

**Figure 2:**
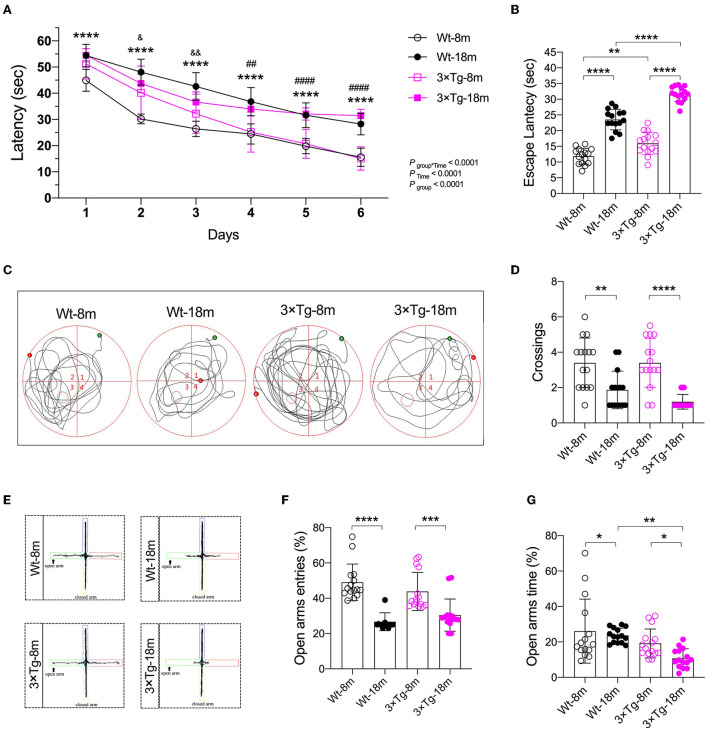
Cognitive impairment during aging in the wild-type and 3×Tg AD mice. **(A)** In the Morris water maze (MWM) test, a learning curve was made after consecutive training. Escape latencies were analyzed using a two-way ANOVA using Tukey's *post hoc* test, *n* = 15, F_(15, 280)_ = 7.493. ^****^P < 0.0001, Wt-18 m vs. Wt-8m; ^##^*P* < 0.05, ^####^*P* < 0.0001, 3×Tg-18 m vs. 3×Tg-8 m; ^&^*P* < 0.05, ^&&^*P* < 0.01, 3×Tg-18 m vs Wt-18 m. **(B–D)** Escape latency [F_(3, 56)_ = 129.2, *P* < 0.001], representative swimming route, and number of crossings [F_(3, 56)_ = 14.25, *P* < 0.001] in the target quadrant were made in the probe test of the MWM test. Data were analyzed with one-way ANOVA using Tukey's *post hoc* test, *n* = 15. **(E–G)** In the elevated plus maze (EPM) test, representative moving route, the number of entries F_(3, 56)_ = 20.43, (*P* < 0.0001), and stay duration [F_(3, 56)_ = 6.277, *P* = 0.0010] in the open arm were recorded. Wt, wild-type mice; 3×Tg, triple transgenic mice. Data are presented as mean ± SEM, n = 15, ^*^P < 0.05, ^**^P < 0.01, ^***^P < 0.001, ^****^P < 0.0001.

The Morris water maze (MWM) test was then performed to evaluate the functional impact of aging on spatial learning and memory. In consecutive training sessions, the mice with different genetic backgrounds successfully located the submerged platform, as indicated by the significant decrease in escape latency (Figure 2A, *P*_time_ < 0.0001). Aged mice from both strains consistently demonstrated longer latency than the middle-aged mice (Figure 2A). The difference between the Wt and 3×Tg-AD mice was observed in the second and third training sessions. In the probe test, the aged mice spent more time locating the submerged platform (11.85 ± 2.53 vs. 23.53 ± 3.27 s in wild-type mice, 15.97 ± 3.57 vs. 31.66 ± 2.38 s in 3×Tg-AD mice; Figures 2B,C) and performed fewer crossings in the target quadrant (3.4 ± 1.4 vs. 1.9 ± 1.1 in wild-type mice, 3.4 ± 1.4 vs. 1.2 ± 0.4 in 3×Tg-AD mice; Figure 2D). One-way ANOVA showed significant differences among the groups in the escape latency and representative swimming route and number of crossings in the target quadrant. A similar performance in recall memory between the two strains of the same age further indicated that spatial memory deficits might be age-related.

To assess the anxiety response, an elevated plus maze test was performed 24 h after the probe test of MWM. Compared with the middle-aged mice, there were consistent decreases in the number of entries and time spent in the open arms in the Wt and 3×Tg-AD aged mice (Figures 2E–G). These data suggested that anxiety existed during aging, particularly in the aged 3×Tg-AD mice.

### Laparotomy-induced inflammation and cognitive impairment in wild-type and 3×Tg-AD mice

**Figure 3:**
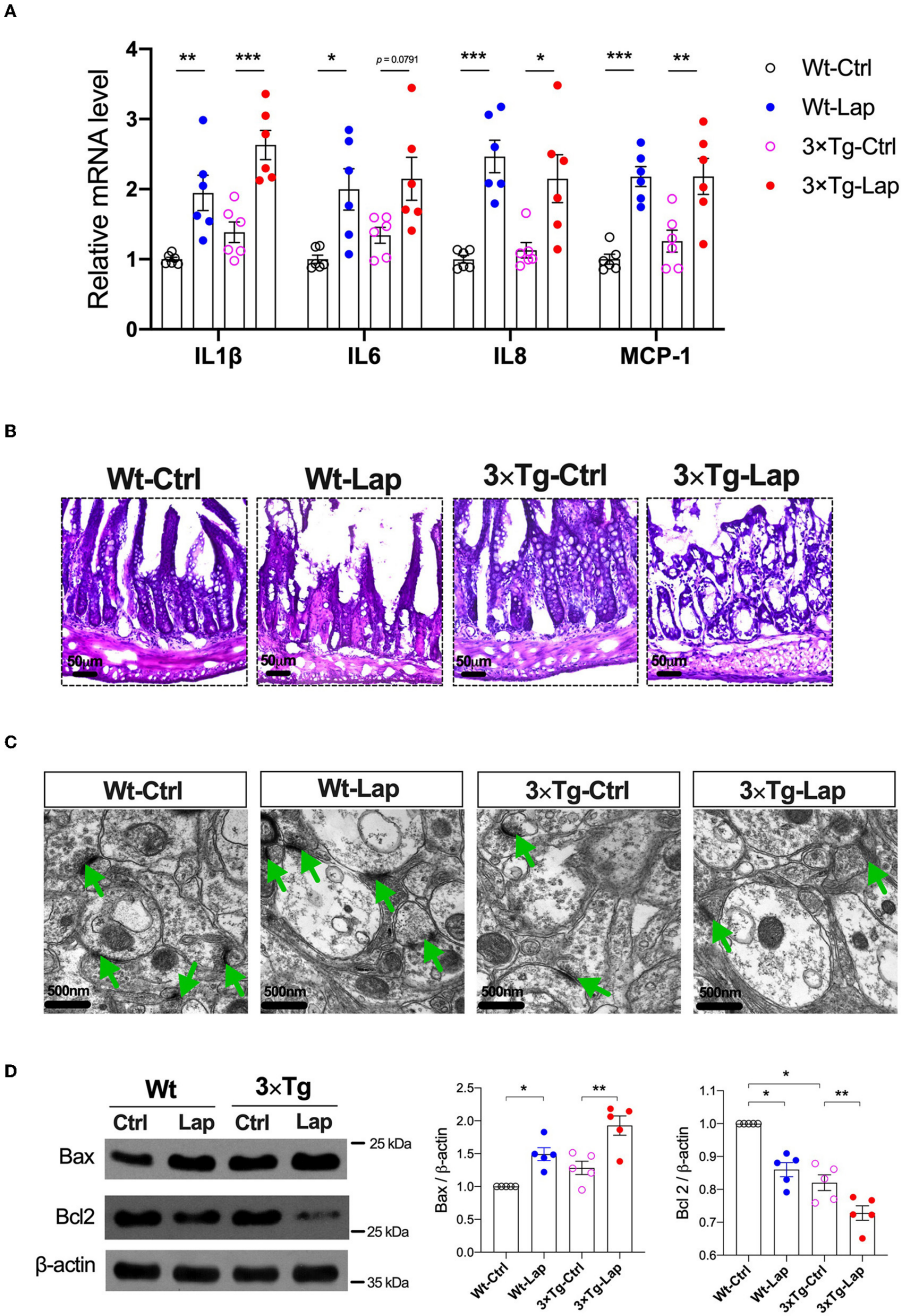
Laparotomy caused similar inflammatory responses in the periphery and hippocampus in both wild-type and 3×Tg AD mice. **(A)** Relative mRNA level of pro-inflammatory cytokines at postoperative 24 h in the hippocampi. IL-1β: F_(3, 20)_ = 15.60, *P* < 0.0001; IL-6: F_(3, 20)_ = 5.97, *P* = 0.0044; IL-8: F_(3, 20)_ = 11.57, *P* = 0.0001; MCP-1: F_(3, 20)_ = 13.18, *P* < 0.0001. **(B)** Morphological changes of acini and base of the intestine in postoperative 14 days by using H&E staining. Representative pictures were obtained from three independent experiments. **(C)** Representative ultrastructure of the hippocampal neuronal synapse using transmission electron microscopy following laparotomy in both wild-type and 3×Tg AD mice. **(D)** Protein expressions of apoptotic proteins, Bax, and Bcl2 in the hippocampi were determined by Western blot analysis. Bax: F_(3, 16)_ = 14.79, *P* < 0.0001; BCL2: F_(3, 16)_ = 33.36, *P* < 0.0001. Wt, wild-type mice; 3×Tg, triple transgenic mice; Ctrl, control; Lap, laparotomy. Data are presented as mean ± SEM and were analyzed using one-way ANOVA using Tukey's *post hoc* test, ^*^P < 0.05, ^**^P < 0.01, ^***^P < 0.001.

Peripheral inflammation and neuroinflammation were observed 24 h postoperatively with increases in the mRNA levels of pro-inflammatory cytokines in the liver (Supplementary Figure S1A) and hippocampus (Figure 3A), respectively. The neuroinflammatory response was still present on postoperative day 14, as indicated by the activation of Iba1^+^ microglia and GFAP^+^ astrocytes, as well as CD68^+^ macrophages observed in the hippocampus (Supplementary Figures S1B,C). Meanwhile, disruption in the intestinal lining and synapses after laparotomy was observed in both strains (Figures 3B,C). There was obvious evidence of apoptosis with a significant increase in Bax (1.49-fold of Wt-Ctrl in wild-type mice, 1.50-fold of 3×Tg-Ctrl in 3×Tg-AD mice) and decrease in Bcl2 (0.86-fold of Wt-Ctrl in wild-type mice, 0.89-fold of 3×Tg-Ctrl in 3×Tg-AD mice) in the hippocampi, and predominant localization of TUNEL^+^ and cleaved caspase 3^+^ cells in the hippocampal CA3 region in both strains following laparotomy (Figure 3D, Supplementary Figure S2).

**Figure 4:**
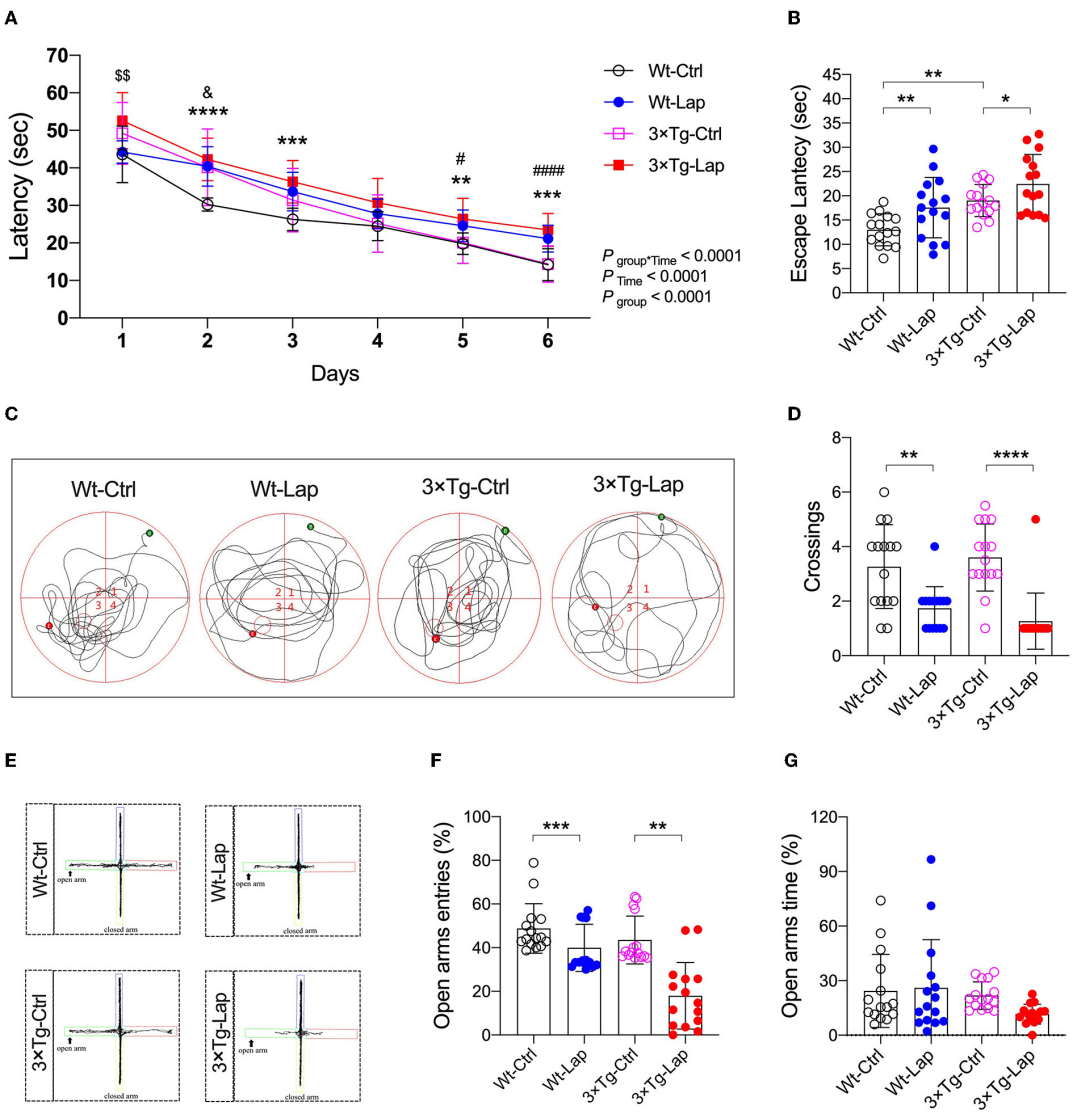
Laparotomy induced cognitive impairment in both wild-type and 3×Tg AD mice. **(A)** In the wild-type and AD mice without or with laparotomy, the learning ability of spatial acquisition was made during the training session in the MWM test. F_(15, 280)_ = 4.066. ^**^P < 0.01, ^***^P < 0.001, ^****^P < 0.0001, Wt-Lap vs. Wt-Ctrl; ^#^*P* < 0.05, ^####^*P* < 0.0001, 3×Tg-Lap vs. 3×Tg-Ctrl; ^&^*P* < 0.05, 3×Tg-Ctrl vs. Wt-Ctrl; ^$$^P < 0.01, 3×Tg-Lap vs. Wt-Lap. **(B–D)** On postoperative day 12 after laparotomy, escape latency [F_(3, 56)_ = 9.531, *P* < 0.0001], representative swimming route, and number of crossings [F_(3, 56)_ = 14.03, P < 0.0001] were recorded in the probe test of the MWM test. **(E–G)** In the EPM test, representative moving tracks, the number of entries [F_(3, 56)_ = 18.44, *P* < 0.0001] and time spent [F_(3, 56)_ =2.098, P = 0.1108] in the open arm were summarized. Wt, wild-type mice; 3×Tg, triple transgenic mice; Ctrl, control; Lap, laparotomy. Data are present as mean ± SEM and were analyzed using one-way ANOVA using Tukey's *post hoc* test, *n* = 15, ^*^P < 0.05, ^**^P < 0.01, ^***^P < 0.001, ^****^P < 0.0001.

All mice successfully acquired spatial learning abilities during the MWM training sessions, although postoperative 3×Tg-AD mice required more time to locate the submerged platform on the first training day. However, the significantly longer escape latency seen on the fifth and sixth training sessions suggested that surgery consistently impaired spatial learning in both wild-type and 3×Tg-AD mice (Figure 4A). During the probe test, the longer escape latency (12.98 ± 3.29 vs. 17.56 ± 6.23 s in wild-type mice, 19.04 ± 3.31 vs. 22.46 ± 6.07 s in 3×Tg-AD mice) and a reduced number of crossings (3.3 ± 1.5 vs. 1.7 ± 0.8 in wild-type mice, 3.6 ± 1.2 vs. 1.3 ± 1.0 in 3×Tg-AD mice) further indicated that surgery could induce memory deficits in both mice strains (Figures 4B–D). The impact of laparotomy on memory in 3×Tg-AD mice was also determined in the Y-maze test. Compared with sevoflurane anesthesia alone, more errors and longer escape latency were observed on postoperative days 1 and 14 in the adult 3×Tg-AD mice. In addition to the body weight loss during the early postoperative period, more errors were observed in the aged 3×Tg-AD mice, rather than a longer escape latency (Supplementary Figure S3). In the elevated plus maze test, anxiety was only presented in postoperative mice, indicated by fewer entries in the open arms (48.81 ± 11.34 vs. 39.93 ± 10.78% in wild-type mice, 43.50 ± 10.95 vs. 17.97 ± 15.27% in 3×Tg-AD mice Figures 4E–G).

### Laparotomy-induced autophagy-dependent PKC activation

**Figure 5:**
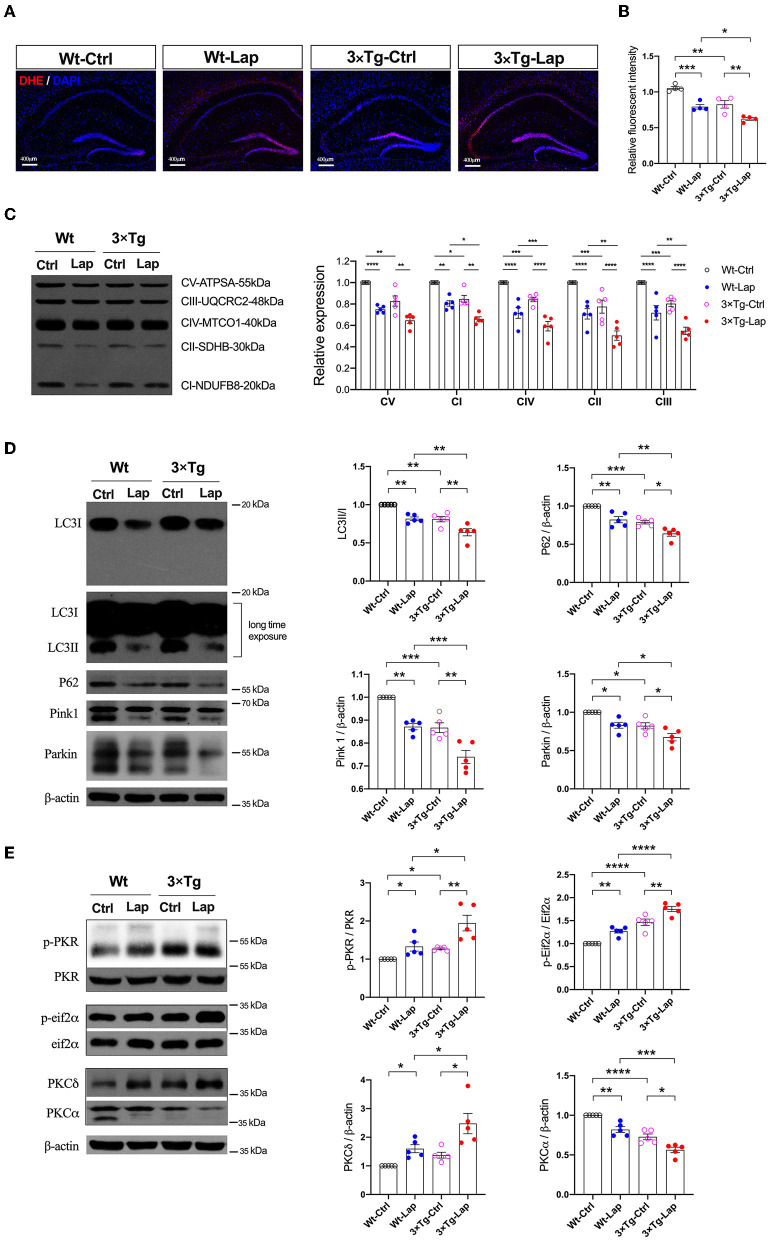
Laparotomy impaired mitochondria mediated by the activation of PKR. **(A)** To evaluate the oxidative stress in the CNS, DHE staining was used in the entire hippocampal region following laparotomy in two strains of animals. Representative pictures were obtained from three independent experiments. **(B)** Mitochondria membrane potentiation was determined by JC-1 assay in the isolated mitochondria fractions in the hippocampi. F_(3, 12)_ = 26.14, *P* < 0.0001. **(C)** After laparotomy, the expression of the mitochondria OXPHOS complex was detected by Western blot analysis in the hippocampi. CV:F_(3, 16)_ = 27.28, *P* < 0.0001; CI: F_(3, 16)_ = 35.95, *P* < 0.0001; CIV:F_(3, 16)_ = 24.85, *P* < 0.0001; CII:F_(3, 16)_ = 20.70, *P* < 0.0001; CIII:F_(3, 16)_ = 21.64, *P* < 0.0001. **(D)** Western blots of autophagy-related proteins, including LC3, P62, Pink1, and Parkin, in the hippocampus following laparotomy in the middle-aged wild-type and 3×Tg AD mice. **(E)** Activities of PKR and PKC in the hippocampus induced by laparotomy in both wild-type and 3×Tg AD mice. Wt, wild-type mice; 3×Tg, triple transgenic mice; Ctrl, control; Lap, laparotomy. Data are presented as mean ± SEM and were analyzed using one-way ANOVA using Tukey's *post hoc* test, ^*^P < 0.05, ^**^P < 0.01, ^***^P < 0.001, ^****^P < 0.0001.

In addition to inducing neuroinflammation, laparotomy can significantly impact mitochondria and autophagy. Consistent with our previous study (Wang et al., 2022), laparotomy caused neuronal oxidative stress in the 3×Tg-AD mice, with significant elevation of ROS, and reduction in the mitochondrial membrane potential (1.053 ± 0.046 vs. 0.793 ± 0.064 in wild-type mice, 0.826 ± 0.106 vs. 0.624 ± 0.040 in 3×Tg-AD mice) and activity of the mitochondrial complexes. In this respect, complexes I, IV, II, and III were significantly decreased after laparotomy in the 3×Tg-AD mice compared with wild-type mice (Figures 5A–C). There were decreases in mitochondrial complex activity in the intact 3×Tg-AD mice compared with the wild-type mice.

At the same time, laparotomy caused consistent decreases in autophagy-related proteins, such as LC3, P62, Pink1, and Parkin, in the hippocampus of both wild-type and 3×Tg-AD mice, especially in the 3×Tg-AD mice (Figure 5D). Compared with the control group, the increased activities in PKR/Eif2α and variations of PKC were significantly expressed in the hippocampus in both strains of mice, with these changes being more pronounced in the postoperative 3×Tg-AD mice (Figure 5E).

**Figure 6:**
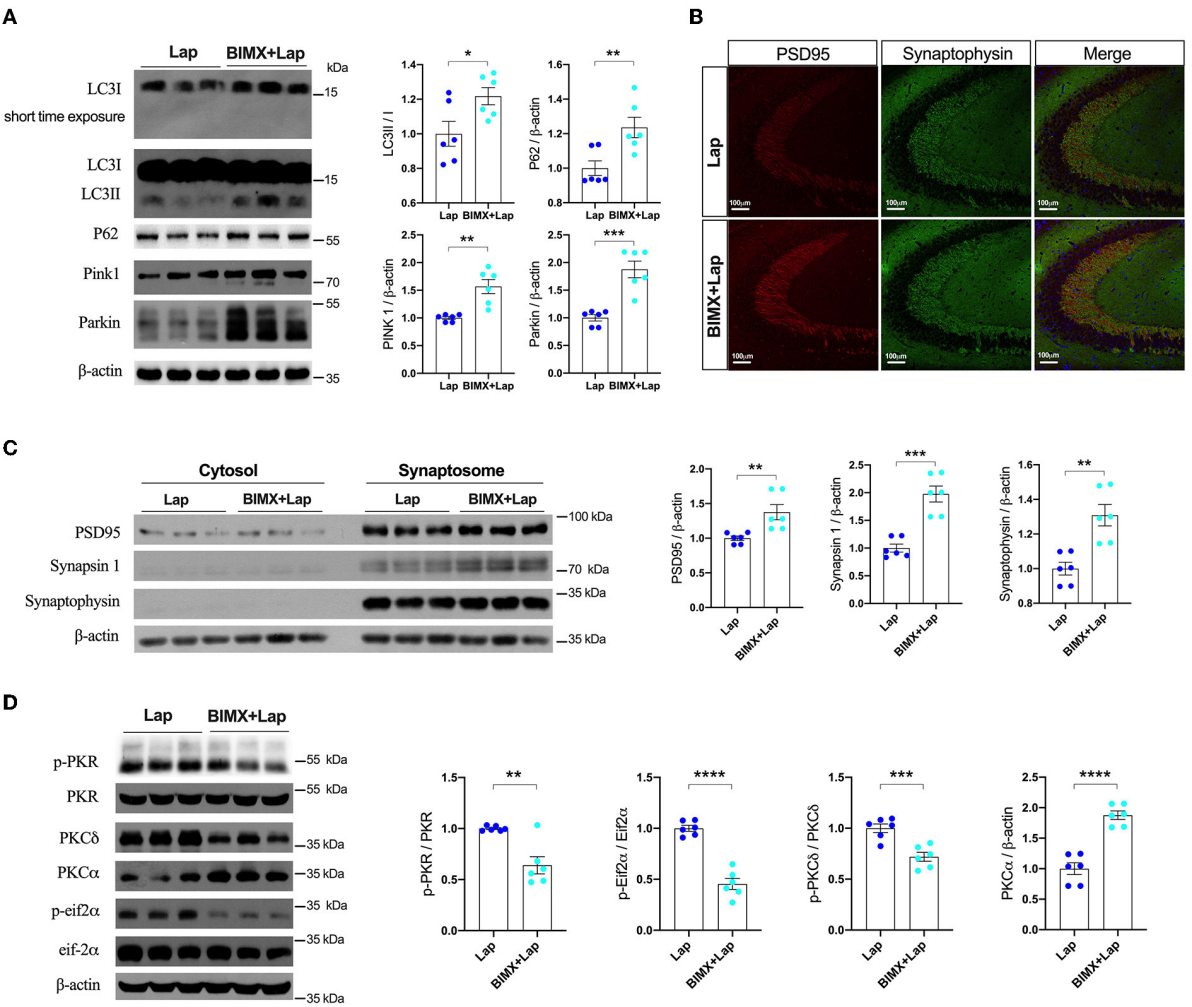
Inhibition of PKC attenuated deficits of autophagy and synapse in middle-aged wild-type mice. **(A)** Autophagy protein expressions without or with PKC inhibitor (BIMX) following laparotomy in the wild-type mice. **(B)** Spread of synaptic proteins, including PSD95 and synaptophysin, was observed using immunofluorescent staining in the cornu ammonis (CA) 3 hippocampal region after laparotomy. **(C)** Synaptic protein expressions in the cytosol and synaptosome in postoperative hippocampi without or with BIMX using Western blot analysis. **(D)** Activities of PKR with pharmacological inhibition of PKC in middle-aged wild-type mice after laparotomy. Wt, wild type; Ctrl, control; Lap, laparotomy. Data are presented as mean ± SEM and were analyzed using one-way ANOVA using Tukey's *post hoc* test, ^*^P < 0.05, ^**^P < 0.01, ^***^P < 0.001, ^****^P < 0.0001.

Pharmacological inhibition of PKC with BIMX resulted in significantly increased expression of autophagy-related proteins after laparotomy (Figure 6A). There were also distinct increases in synaptic proteins such as PSD95 (1.375-fold of Lap), synapsin 1 (1.976-fold of Lap), and synaptophysin (1.309-fold of Lap) in the synaptosome fractions in the hippocampal CA3 region (Figures 6B,C). Furthermore, neuronal apoptosis was attenuated by BIMX, with significant decreases in Bax and cleaved caspase-3 expression (Supplementary Figure S4). The activities of PKR/Eif2α and PKCδ were also decreased with an increase in PKCα in the hippocampi of the 3×Tg-AD mice (Figure 6D).

### Laparotomy-induced autophagy-dependent PKR activation

**Figure 7:**
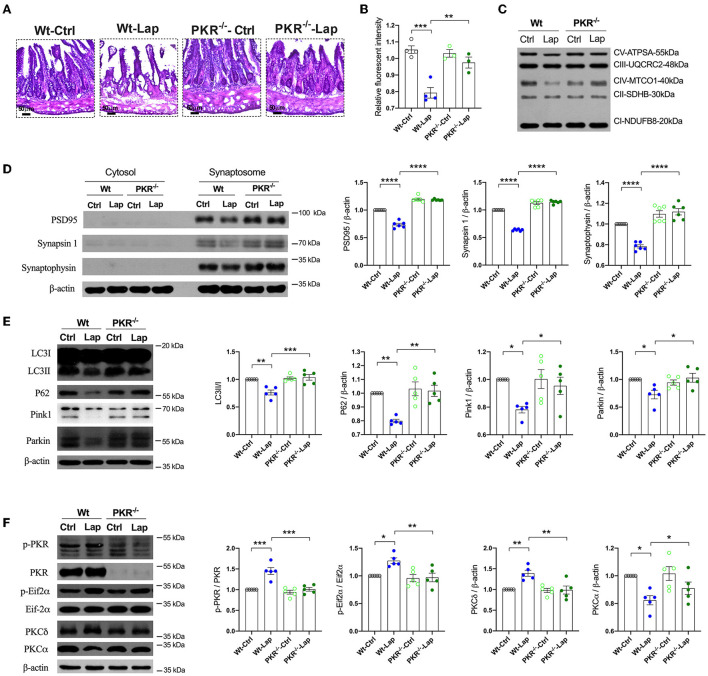
Pathological signatures induced by laparotomy were determined by PKR activity. **(A)** Representative morphological changes of the intestine by H&E staining following laparotomy in the wild-type and PKR^−/−^ mice. **(B)** Mitochondria membrane potentiation was detected by JC-1 assay in the isolated mitochondria fractions. F_(3, 10)_ = 18.96, *P* = 0.0002. **(C)** Representative Western blots of the mitochondria OXPHOS complex in the hippocampi after laparotomy in the wild-type and PKR^−/−^ mice. **(D)** Western blots of synaptic proteins in the isolated synaptosome from postoperative hippocampi in the wild-type and PKR^−/−^ mice. **(E)** Relative expressions of autophagy proteins, LC3B: F_(3, 16)_ = 12.58, *P* = 0.0002; P62: F_(3, 16)_ = 21.14, *P* < 0.0001; Pink1: F_(3, 16)_ = 17.26, *P* < 0.0001; Parkin: F_(3, 16)_ = 43.14, *P* < 0.0001. **(F)** Relative activities of PKC induced by laparotomy in the wild-type and PKR^−/−^ mice. Wt, wild type; Ctrl, control; Lap, laparotomy. Data are presented as mean ± SEM and were analyzed using one-way ANOVA using Tukey's *post hoc* test, ^*^P < 0.05, ^**^P < 0.01, ^***^P < 0.001, ^****^P < 0.0001.

To further investigate the role of PKR in the neuropathological changes induced by laparotomy, the PKR^−/−^ mice were subjected to laparotomy under sevoflurane anesthesia. The disruption in the intestinal lining, increases in hippocampal ROS, and the impairment of mitochondrial membrane potentiation seen postoperatively in wild-type and AD mice were not observed in the PKR^−/−^ mice (Figures 7A–C). The decreases in synaptic proteins (PSD95, synapsin 1, and synaptophysin) and autophagy-related proteins (LC3, P62, Pink1, and Parkin) observed in the wild-type and AD mice were not present after genomic silencing of PKR (Figures 7D,E, Supplementary Figure S6). Furthermore, PKR/Eif2α and PKC activities were not changed in the postoperative PKR^−/−^ mice (Figure 7F). Finally, the PKR^−/−^ mice that underwent laparotomy and control mice exhibited a comparable escape latency during the MWM test (Supplementary Figure S7).

## Discussion

It is well established that clinically older patients and those with preexisting cognitive impairment are more susceptible to developing PND. In this respect, it has been shown that in patients aged 60 years or older undergoing elective total hip replacement, a low CSF Aβ_1−42_ may be a significant predictor of PND at 3 months postoperatively (Evered L. et al., 2016). Advanced age may independently affect cognition by reducing PKCα/ε expression mediated by PKC/HuD/BDNF signaling, leading to alterations in inhibitory synaptic transmission and reduced mushroom spine synapses (Hongpaisan et al., 2013). Using murine models of Alzheimer's disease and perioperative neurocognitive disorders, we compared the neuropathological characteristics of these two conditions in the mice of different ages. In addition to the inflammatory changes in the periphery and central nervous system, mitochondrial dysfunction, defective autophagy, synaptic dysfunction, and neuronal apoptosis were documented. Importantly, the unique roles of PKC/PKR in these changes were examined.

Our study substantiated the adverse impact of aging on cognitive impairment in the wild-type and 3×Tg-AD mice. Similar to 3-month-old young wild-type mice with better learning ability compared with APPswe/PS1dE9 mice (Jiang et al., 2018), aged wild-type mice (18 months old) also required a shorter time to find the hidden platform than the aged 3×Tg-AD mice in the Morris water maze test. Aging significantly impacts cognition levels after surgery. Another study demonstrated that aged mice (19 months old) and young surgical mice exhibited comparable cognitive performance in the Barnes maze test (Lai et al., 2021). In animals, it has been shown that PND could be triggered by neuroinflammation in peripheral surgery models such as left carotid artery exposure (Lai et al., 2021) and laparotomy (Huang et al., 2018). In the wild-type and 3×Tg-AD mice of the same age, laparotomy induced PND with significant intestinal disruption and glial activation. However, although partial hepatectomy significantly exacerbated cognitive deficits in the APPswe/PS1dE9 transgenic mice (Jiang et al., 2018), laparotomy failed to replicate this finding in the middle-aged 3×Tg-AD mice in our study (Figure 4).

It is widely acknowledged that autophagy is initiated to restore neurotransmitter release, synaptic plasticity, and neuronal survival (Limanaqi et al., 2021). As a specialized form of autophagy, defective mitophagy is critical in the development and progression of AD, according to cross-species analyses of both transgenic animal models and human patients with AD (Swerdlow et al., 2014; Kerr et al., 2017). In this study, we provided compelling evidence that advanced age and surgery are associated with mitochondrial dysfunction and defective autophagy in both wild-type and 3×Tg-AD mice. Laparotomy had a greater impact on the mitochondria and autophagy in the 3×Tg-AD mice than in the wild-type mice. In AD, multiple mitophagy and autophagy pathways related to AD mitochondrial cascade hypothesis have been documented (Lazarou et al., 2015; Menzies et al., 2015; Kerr et al., 2017). Current evidence suggests that both Pink1 (Du et al., 2017) and Parkin (Khandelwal et al., 2011) improve mitochondrial functions and eliminate intracellular Aβ. Importantly, the regulation of autophagy depends on the activity of PKC in association with the P13K/Akt/mTOR signaling pathway (Harris et al., 2020). Although PKCs are particularly sensitive to redox stress (Giorgi et al., 2010), lysosomal activities are increased through the PKC–transcription factor EB (TFEB) axis even under nutrient-rich conditions (Li et al., 2016). To eliminate nonfunctional protein aggregations, autophagy activators can induce autophagy *via* the PRKC/PKC–TFEB pathway in cellular models of Parkinson's disease and Huntingdon disease (Kataura et al., 2021).

In aged mice, activation of PKC has been shown to reverse age-related neuropathological changes in the hippocampal neurons to regenerate memory comparable to young rats (Hongpaisan et al., 2013). Indeed, in the AD brain, levels of PKC, its receptors, activity, and associated phosphatases are decreased (Harris et al., 2020). Aβ has been shown to directly reduce PKC protein levels and activity and attenuate kinase translocation to the cell membrane in AD brains (Wang et al., 1994) and in *in vitro* studies (Lee et al., 2004). During APP processing, different PKC isoforms act on different cleavage sites to induce both amyloidogenic and nonamyloidogenic cleavages (Lanni et al., 2004; Galvao et al., 2019). Studies on AD have shown that PKC activation has a positive impact on amyloid pathology, enhancing the secretion of the α-secretase product sAPPα and reducing Aβ40 (Etcheberrigaray et al., 2004) and tau pathology (Isagawa et al., 2000). On the other hand, inhibition of PKC and NOX can strongly attenuate the direct neurotoxicity of Aβ and the effects on lysosomal and mitochondrial functions in the hippocampal neurons (Li and Jiang, 2018). We found consistent increases in PKCδ expression in the wild-type and 3×Tg-AD mice with either aging or laparotomy. The increases in PKCδ were reinforced in the 3×Tg-AD mice following laparotomy. The inhibition of PKC by BIMX dramatically attenuated the effects of laparotomy on autophagy and mitochondrial functions.

PKCα is one of the three highly penetrant variants in the PRKCA gene, identified by whole-genome sequencing of 1345 individuals from 410 families with late-onset AD (Alfonso et al., 2016). Intriguingly, an AD-associated mutation in PKCα permits enhanced agonist-dependent signaling *via* evading the cell homeostatic downregulation of constitutively active PKCα in AD (Callender et al., 2018). Interestingly, PKCα contributes to tau phosphorylation in the early stages of frontotemporal lobar degeneration (Fujita et al., 2018) and synaptogenesis. Moreover, the actions of Aβ on synapses are reportedly mediated by PKCα through scaffold interactions (Alfonso et al., 2016). In the initial stages of AD, accumulated intracellular amyloid-beta oligomer causes functional spreading of hyperexcitability through a synaptic-driven mechanism, which is PKC-dependent (Fernandez-Perez et al., 2021). Significant suppression of PKCα activity was observed in the wild-type and 3×Tg-AD mice in the aged or postsurgical hippocampus. This suppressive effect induced by laparotomy was enhanced in the 3×Tg-AD mice. The decrease in synaptic proteins following laparotomy was successfully rescued by the inhibition of PKC with BIMX.

PKR is a pro-apoptotic kinase that inhibits protein translation that has been implicated in several molecular pathways that lead to AD brain lesions and disturbed memory formation and consolidation (Hugon et al., 2017). PKR is also required for autophagy stimulation and accumulation (Ogolla et al., 2013). Given that significant elevations in total PKR and p-PKR concentrations have been documented in AD and amnestic mild cognitive impairment subjects, the assessment of T-PKR and p-PKR in cerebrospinal fluid could be used to diagnose AD (Mouton-Liger et al., 2012). A study reported that the cognitive scores of 299 participants exhibited a significant negative relationship with the protein expressions of IRS1, JNK, and PKR in their neocortex. These proteins are also significantly associated with the pathological brain signatures in'patients with Alzheimer's disease (Taga et al., 2017). Moreover, in AD animal models, either β-amyloid oligomers or AD-associated toxins initiate the elevation of phosphorylated Eif2α, neuronal insulin receptor substrate inhibition, synapse loss, and cognition impairment through activating PKR (Lourenco et al., 2013). Furthermore, PKR initially facilitated tau hyperphosphorylation, which is independent of PKR-mediated downstream activation of GSK-3β or neuroinflammation in AD and other tauopathies (Reimer et al., 2021). In our previous study, PKR activation positively regulated autophagy and tau phosphorylation in PND induced by laparotomy in aged wild-type mice (Wang et al., 2022). Therefore, significant improvement in cognitive deficits was achieved when PKR activity was suppressed with a selective PKR inhibitor (SAR439883). Meanwhile, there were prominent decreases in synaptic protein loss, pro-inflammatory cytokine accumulation, and neurodegeneration in AD experimental mice with ApoE4 overexpression or intracerebroventricular Aβ-injection (Lopez-Grancha et al., 2021). When PKR activity was silenced by using a genomic approach, such as double-mutant 5×FAD with PKR^−/−^, 9-month-old mice displayed significant improvements in memory consolidation and a series of neuropathological changes in the brain as compared to 5×FAD mice, including decreases in amyloid accumulation, BACE1 expression, neuronal apoptosis, neurodegeneration markers, and synaptic alterations, as well as a neuroinflammatory burden. This finding further corroborates the indispensable contribution of PKR to microglia-induced neuronal death in neuron-glial cocultures after LPS stimulation (Tible et al., 2019). In this study, we substantiated the role of PKR in aging and PND in 3×Tg-AD mice. There were significant increases in PKR activity by phosphorylation in the hippocampus. However, neuropathological variations induced by laparotomy were not observed in PKR^−/−^ mice.

A major limitation of this study is that we did not examine the effect of gender. It is well established that estradiol induces gene transcription and rapid membrane signaling mediated by three receptors, including estrogen receptor-alpha (ERα), estrogen receptor-beta (ERβ), and a recently characterized G-protein-coupled estrogen receptor. Moreover, studies using receptor-specific agonists have shown that all three receptors rapidly activate kinase signaling and have complex dose-dependent influences on memory (Bean et al., 2014). The binding of endogenous estradiol (E2) and ERs affects hippocampal learning and memory function by activating various cell signaling kinases, such as phosphatidylinositol 3-kinase (PI3K), extracellular signal-regulated kinase (ERK), protein kinase A (PKA), and PKC (Fan et al., 2010). In a transgenic model of Alzheimer's disease, female mice exhibited more extensive amyloid lesions, but not tau lesions (Rae and Brown, 2015). Mortality in 3×Tg-AD mice appeared as early as 12 months since tau lesions developed at 9-12 months. 3×Tg-AD male mice exhibited earlier mortality than female mice. Accordingly, tau pathology may be more aggressive in male than in female mice (Hirata-Fukae et al., 2008; Rae and Brown, 2015). Although the prevalence of the disease is higher in female mice, neuroimmunoendocrine factors may cause early mortality in male mice. The higher vulnerability of the neuroimmunoendocrine network in male mice could result in a higher susceptibility to deleterious effects of aging and be responsible for the increased morbidity and mortality observed in 3×Tg-AD male mice (Clinton et al., 2007; Gimenez-Llort et al., 2008). In this study, only male mice were used to exclude the influence on E2 on the areas of focus. Nonetheless, future studies should compare the effect of gender on these findings.

## Conclusions

Aging, AD, and PND appear to share similar pathogenic mechanisms, including altered mitochondrial homeostasis and neuroinflammation. Laparotomy consistently caused cognitive deficits accompanied by peripheral and neuronal inflammation, mitochondrial dysfunction, synaptic proteins, and neuronal loss in wild-type and 3×Tg-AD mice. The inhibition of PKC and PKR may be the key to developing new drug candidates for aging, AD, and PND therapy.

## Data availability statement

The datasets presented in this study can be found in online repositories. The names of the repository/repositories and accession number(s) can be found in the article/Supplementary material.

## Ethics statement

All experimental protocols and animal handling procedures were approved by the Faculty Committee on the Use of Live Animals in Teaching and Research in The University of Hong Kong (CULATR Ref. No. 3437-14) and the Ethics Committee for the use of experimental animals in Anhui Medical University (reference number LLSC 20190765; date of approval 26/10/2019).

## Author contributions

WL and ST performed the experiments and data acquisitions. AL, QH, and MD contributed to the interpretation of data. YZ, XH, and RC helped prepare the manuscript. GW contributed to the experimental design and manuscript preparation. CH designed the study, performed data analysis, and prepared the manuscript. All authors contributed to the article and approved the submitted version.

## Funding

This study was supported by the National Natural Science Foundation of China [Grant Number 81801050], Basic and Clinical Cooperative Research Promotion Plan of Anhui Medical University [Grant Number 2019xkjT026], Scientific Research Platform Base Construction and Promotion Project of Anhui Medical University [Grant Number 2020xkjT060], and NSFC Incubation Project of the Second Hospital of Anhui Medical University [Grant Number 2019GQFY14]. The experiments carried out in Hong Kong on PKR were supported by General Research Fund 17123217 to RC.

## Conflict of interest

The authors declare that the research was conducted in the absence of any commercial or financial relationships that could be construed as a potential conflict of interest.

## Publisher's note

All claims expressed in this article are solely those of the authors and do not necessarily represent those of their affiliated organizations, or those of the publisher, the editors and the reviewers. Any product that may be evaluated in this article, or claim that may be made by its manufacturer, is not guaranteed or endorsed by the publisher.

## Abbreviations

PND: perioperative neurocognitive disorder
POCD: postoperative cognitive dysfunction
AD: Alzheimer's disease
PKC: protein kinase C
CNS: central nervous system
PKR: double-stranded RNA (dsRNA)-dependent protein kinase
APP: β-amyloid precursor protein
OCT: optimal cutting temperature
MCP-1: monocyte chemoattractant protein-1
ROS: reactive oxygen species
DHE: dihydroethidium
BIMX: bisindolylmaleimide X
MWM: Morris water maze
TFEB: transcription factor EB
E2: endogenous estradiol
PI3K: phosphatidylinositol 3-kinase
ERK: extracellular signal-regulated kinase
PKA: protein kinase A.
