# Supplementary material for: The role of PKC/PKR in aging, Alzheimer's disease, and perioperative neurocognitive disorders
Source: Front Aging Neurosci. 2022 Sep 12;14:973068. doi: 10.3389/fnagi.2022.973068 (PMC9510619; doi:10.3389/fnagi.2022.973068)
Supplement: Supplementary file 1 [file Data_Sheet_1.docx]

**Supplementary Materials**

Figure S1: Laparotomy induced periphery inflammation and neuroinflammatory responses in middle aged wild type and 3×Tg AD mice.

Figure S2: Laparotomy induced neuronal apoptosis in middle aged wild type and 3×Tg AD mice.

Figure S3: Laparotomy caused memory deficits in middle aged and aged 3xTg AD mice.

Figure S4: Apoptosis was attenuated by the inhibition of PKC in middle aged 3×Tg AD mice.

Figure S5: Quantified analysis of OXPHOS complex in the isolated mitochondria fractions from the hippocampi. Laparotomy induced neuronal apoptosis in middle aged wild type and 3×Tg AD mice.

Figure S6: Laparotomy changed synaptic protein presentation in the hippocampus in wild type and PKR-/-mice

Figure S7: Laparotomy failed to cause learning deficit in the middle-aged PKR^-/-^ mice.


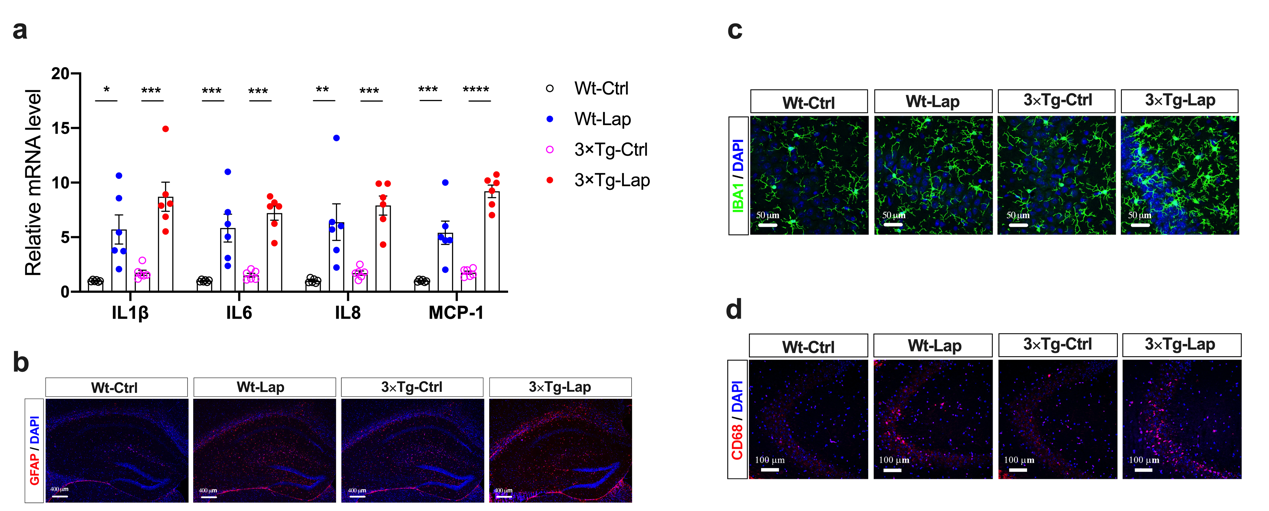


**Figure S1: Laparotomy induced peripheral and neural inflammatory responses in the hippocampus in middle aged wild type and 3×Tg AD mice.**

(**a**) Relative mRNA level of pro-inflammatory cytokines by QT-PCR in the liver. Laparotomy significantly promoted the elevation of pro-inflammatory cytokines at 24 hours. IL-1β: F_(3, 20)_ = 14.30, *P* < 0.0001; IL-6: F_(3, 20)_ = 18.43, *P* < 0.0001; IL-8: F_(3, 20)_ = 12.91, *P* < 0.0001; MCP-1: F_(3, 20)_ = 37.91, *P* < 0.0001. Data present as mean ± SEM, and were analyzed by using one-way ANOVA analysis with Tukey post hoc test, n = 6, ^*^*P* < 0.05, ^**^*P* < 0.01, ^***^*P* < 0.001, ^****^*P* < 0.0001.

(**b-c**) By using immunofluorescent staining, the activation of microglia and astrocyte following laparotomy was observed under con-focal microscopy. In the postoperative 14 days, laparotomy caused the activated GFAP^+^ astrocytes spreading in the entire hippocampus. In particular, IBA1 labelled microglia as well as CD68^+^ cells were also activated in the hippocampal CA3 region. Wt, wild type mice; 3×Tg, triple transgenic mice; Ctrl, Control; Lap, Laparotomy.


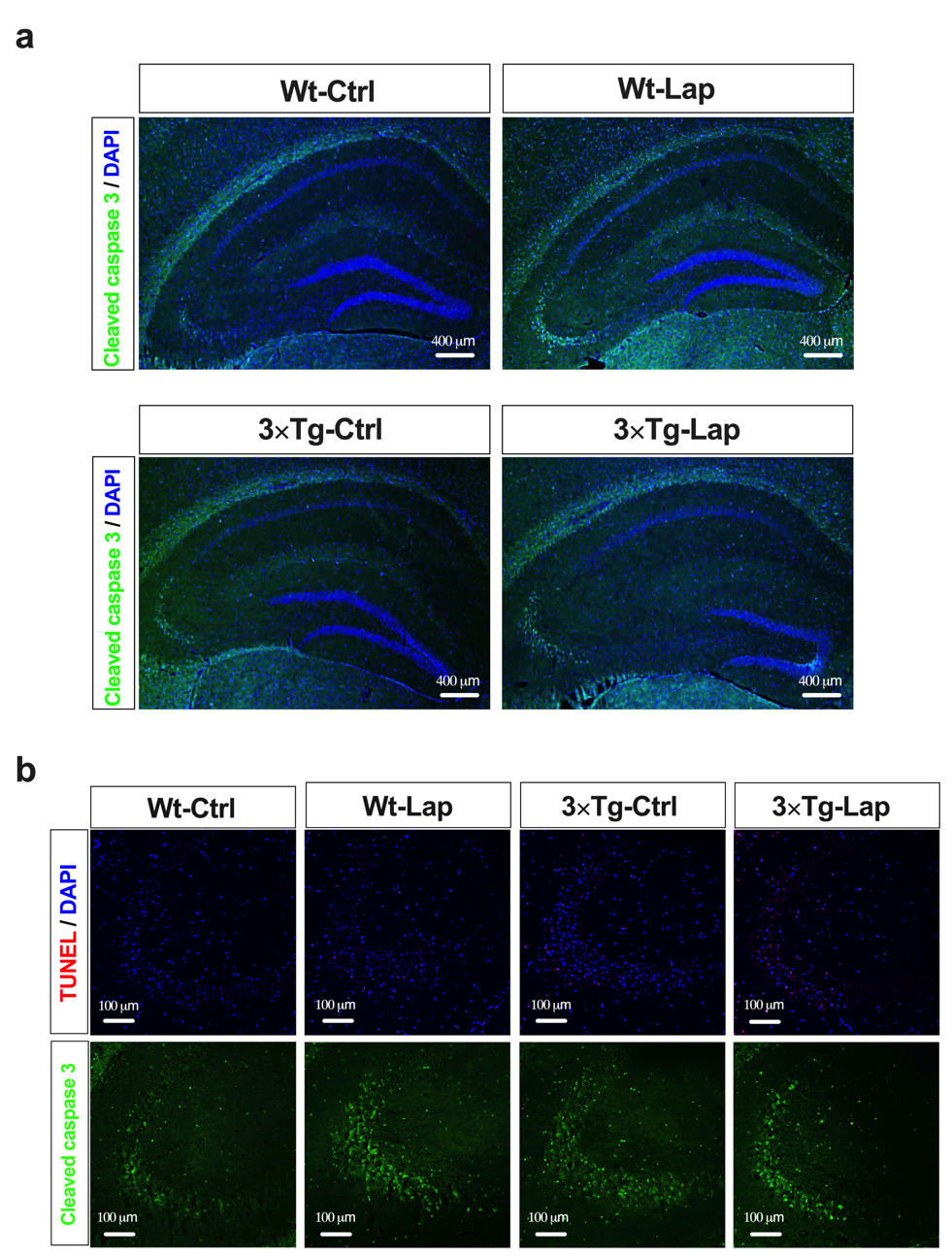


**Figure S2: Laparotomy induced neuronal apoptosis in middle aged wild type and 3×Tg AD mice.**

Laparotomy caused obvious apoptosis concentrating in the hippocampal CA3 region. Then apoptosis was further observed under con-focal microscopy by using immunofluorescent staining with TUNEL and cleaved caspase 3 labelling in the hippocampal CA3 region. Wt, wild type; 3×Tg, transgenic AD; Ctrl, Control; Lap, Laparotomy.


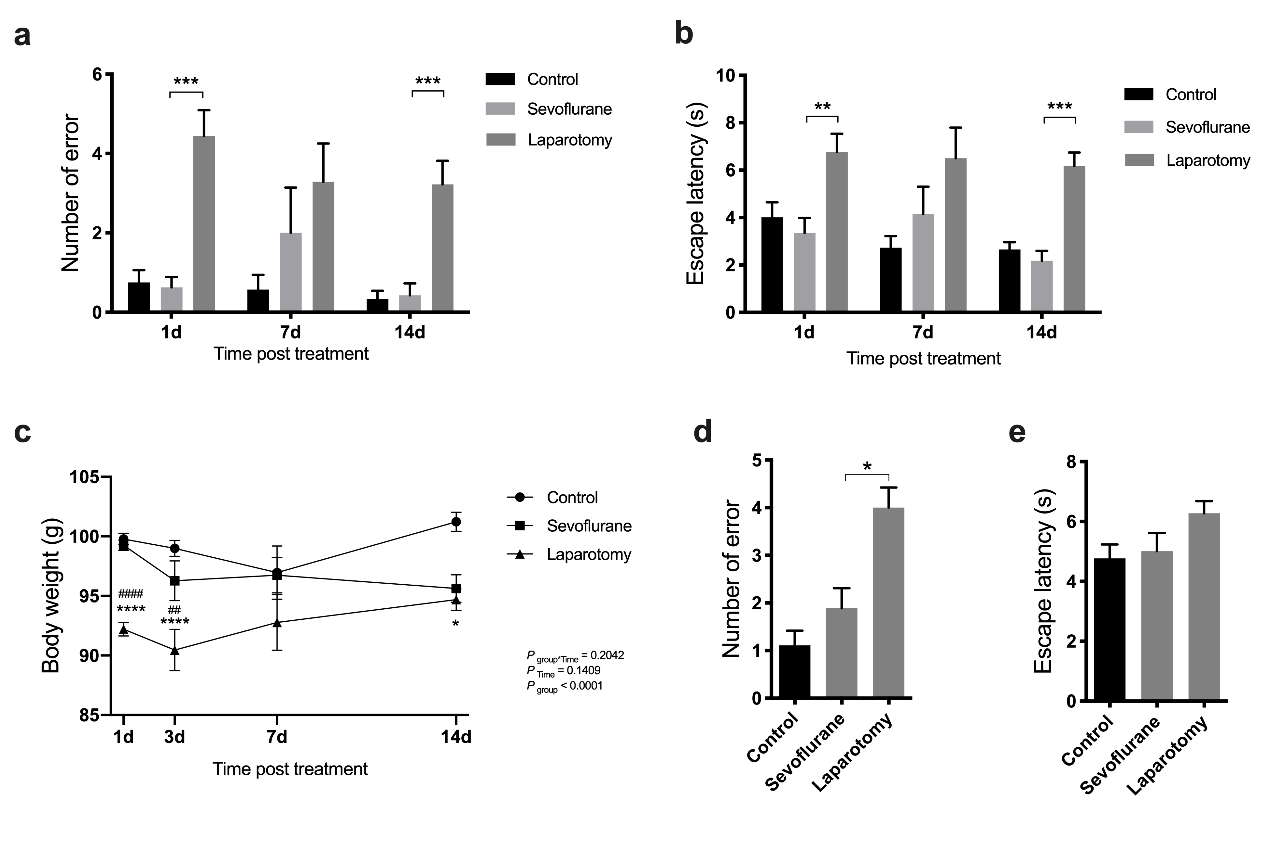


**Figure S3: Laparotomy caused memory deficits in** **middle aged and aged 3×Tg AD mice.**

(**a-b**) To determine the effect of sevoflurane and laparotomy on cognition in middle aged 3×Tg AD mice, Y-maze tests were performed on postoperative day (POD) 1, 7 and 14, respectively. Detail about the Y-maze training and test were described in our previous study [1]. Data present as mean ± SEM, and were analyzed by using one-way ANOVA analysis with Tukey post hoc test or Kruskal-Wallis test. Error number and escape latency were two indicators evaluating the cognition. For number of error, F_24h_ = 16.41, *P* < 0.0001; Kruskal-Wallis statistic_7d_ = 4.525, *P* = 0.0982; F_14d_ = 17.38, *P* < 0.0001. For escape latency, F_24h_ = 5.228, *P* = 0.0027; Kruskal-Wallis statistic_7d_ = 4.401, *P* = 0.1088; F_14d_ = 20.04, *P* < 0.0001. ^**^*P* < 0.01, ^***^*P* < 0.001, n = 10.

(**c**) In aged 3×Tg AD mice (18 months old), body weight as percentage of baseline before the treatment was evaluated at four different postoperative time points (two-way ANOVA analysis with Tukey post hoc test). ^*^*P* < 0.05, ^****^*P* < 0.0001 v.s. Control; ^##^*P* < 0.01, ^####^*P* < 0.0001 v.s. Sevoflurane.

(**d-e**) The performance of aged 3×Tg AD mice (18 months old) in Y-maze test at 14 days following sevoflurane anesthesia without or with laparotomy. Data present as mean ± SEM., n = 8-10, ^*^*P* < 0.05.


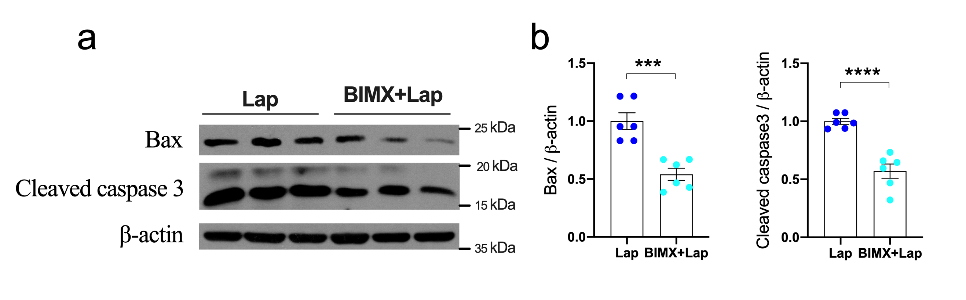


**Figure S4: Apoptosis was attenuated by the inhibition of PKC in** **middle aged 3×Tg AD mice.**

By using western blotting analysis, apoptotic proteins expressions were evaluated in the hippocampi after laparotomy without or with BIMX administration. Data present as mean ± SEM and analyzed with unpaired *t* test, ^***^*P* < 0.001, ^****^*P* < 0.0001. Bax: *t* = 5.182, *P* = 0.0004; Cleaved caspase 3: *t* = 6.469, *P* < 0.0001. Lap, Laparotomy; BIMX, PKC inhibitor.


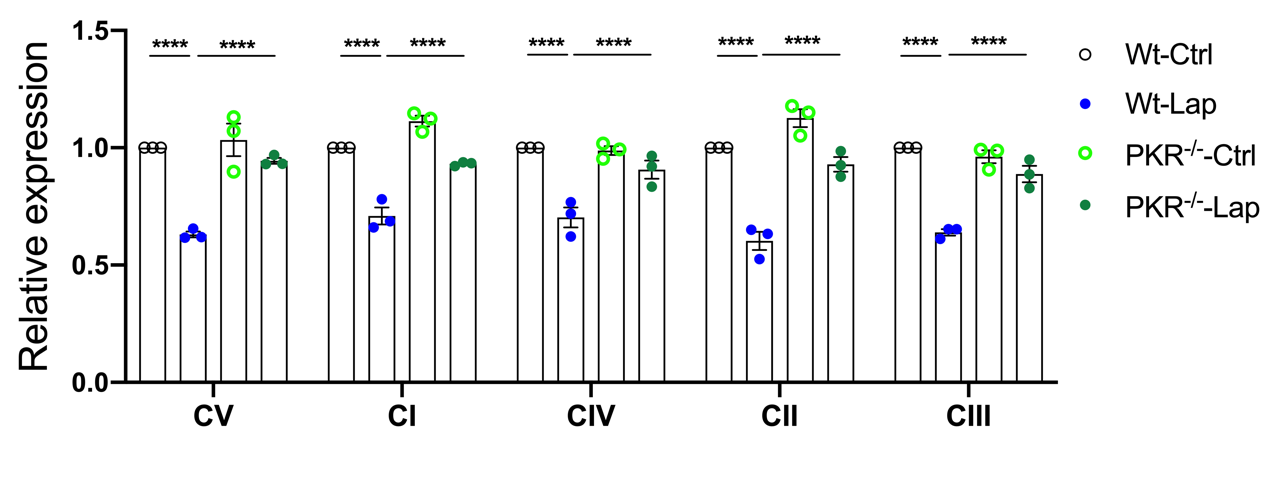


**Figure S5: Quantified analysis of OXPHOS complex in the isolated mitochondria fractions from the postoperative hippocampi in both wild type and PKR^-/-^ mice.**

Relative activities of OXPHOS were determined by using western blot analysis, and were analyzed by one-way ANOVA followed by the Tukey *post hoc* test, CV: F_(3, 8)_ = 26.52, *P* = 0.0002; CI: F_(3, 8)_ = 61.12, *P* < 0.0001; CIV: F_(3, 8)_ = 20.31, *P* = 0.0004; CII: F_(3, 8)_ = 50.24, *P* < 0.0001; CIII: F_(3, 8)_ = 48.41, *P* < 0.0001. Data present as mean ± SEM., ^****^*P* < 0.0001. Wt, wild type; Ctrl, Control; Lap, Laparotomy.


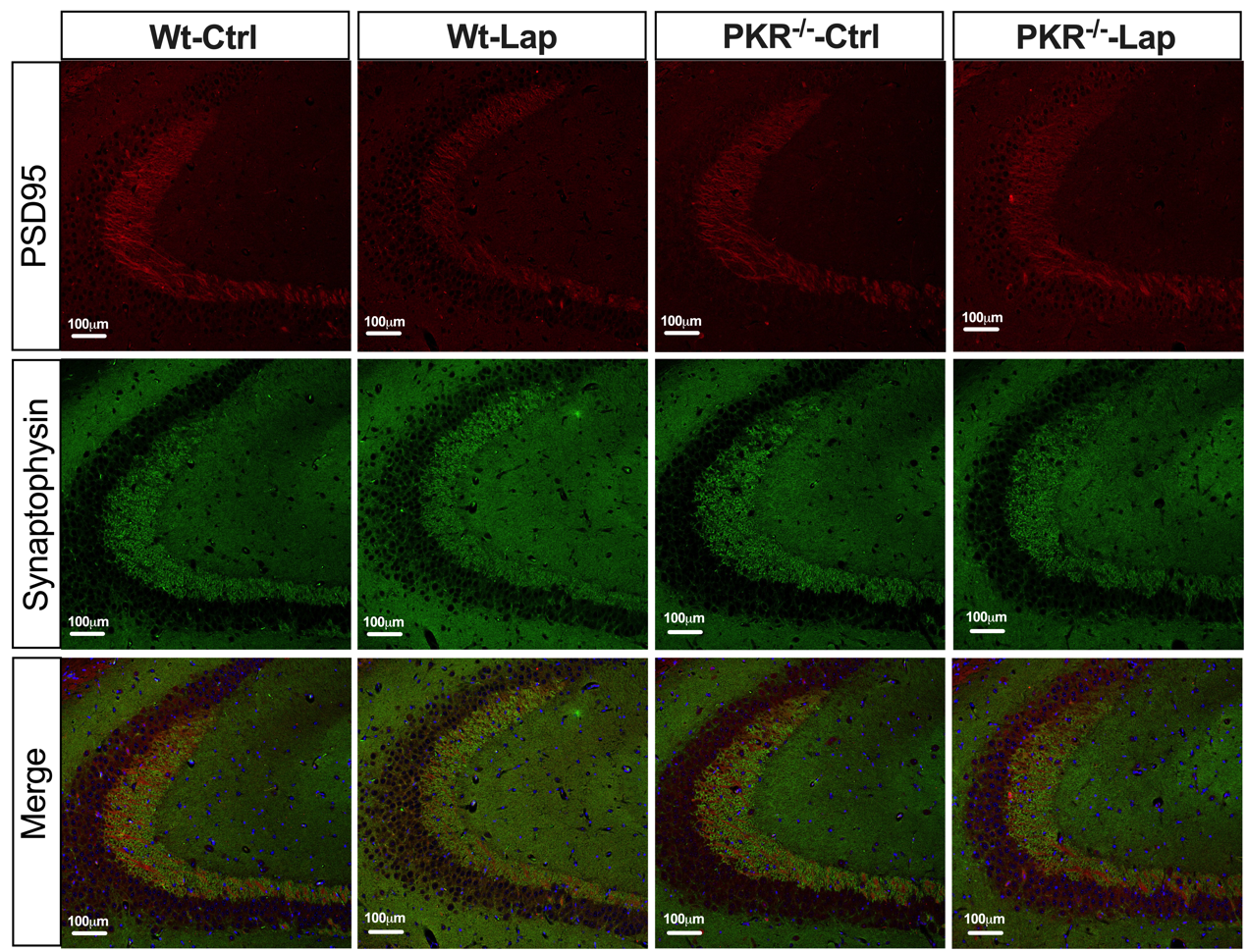


**Figure S6: Laparotomy changed synaptic protein presentation in the hippocampus in wild type and PKR-/-mice**

By using immunofluorescent staining, post-synaptic PSD95 and pe-synaptic Synaptophysin were observed under con-focal microscopy. Wt, wild type; Ctrl, Control; Lap, Laparotomy.


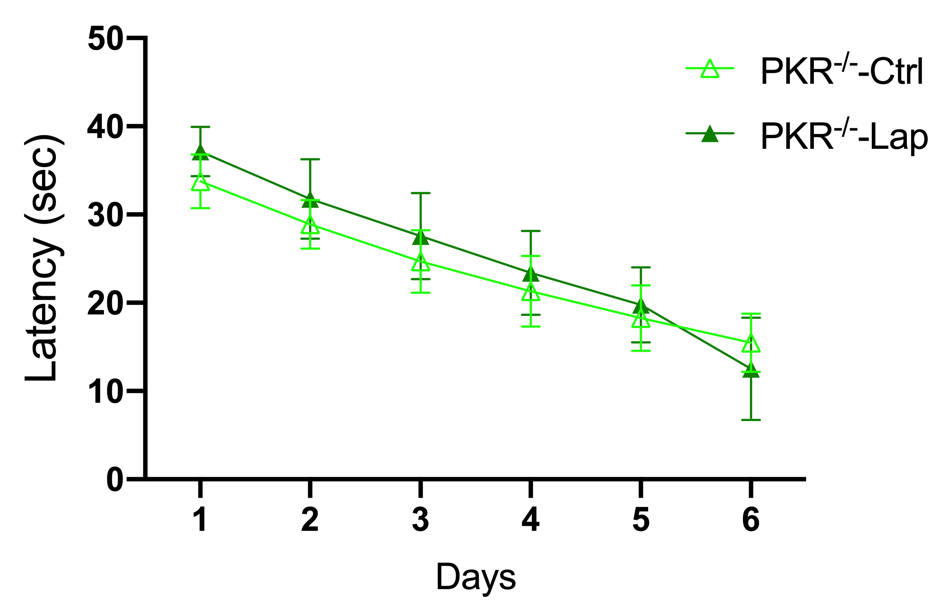


**Figure S7: Laparotomy failed to cause learning deficit in the** **middle-aged PKR^-/-^ mice.**

In the Morris Water Maze test, learning curve was made after consecutive training from postoperative day 7 to 12. Ctrl, Control; Lap, Laparotomy.

**Reference**

[1] Huang C, Irwin MG, Wong GTC, Chang RCC. Evidence of the impact of systemic inflammation on neuroinflammation from a non-bacterial endotoxin animal model. Journal of neuroinflammation. 2018;15:147.
